# A decrease in taxonomic and functional diversity of dung beetles impacts the ecosystem function of manure removal in altered subtropical habitats

**DOI:** 10.1371/journal.pone.0244783

**Published:** 2021-01-06

**Authors:** Rodrigo Sarmiento-Garcés, Malva Isabel Medina Hernández

**Affiliations:** Departamento de Ecologia e Zoologia, Centro de Ciências Biológicas, Universidade Federal de Santa Catarina, Florianópolis, Santa Catarina, Brasil; Institute of Systematics and Evolution of Animals Polish Academy of Sciences, POLAND

## Abstract

The loss of biodiversity—caused mainly by habitat destruction—is one of the environmental problems with major repercussions on ecosystem functioning. Nevertheless, our understanding of the functional consequences of habitat changes on the communities and ecosystems remains limited to a small number of case studies. We evaluated the change in taxonomic and functional diversity of copro-necrophagous beetles (Scarabaeinae) and their relationship with the varying environmental factors present in four habitats with different degrees of disturbance. Furthermore, we evaluated how changes in taxonomic and functional diversity affect the rates of excrement removal. The collections were carried out at four locations in the state of Santa Catarina, Southern Brazil, on natural systems with different degrees of disturbances (forests in advanced and initial succession) and agroecosystems (silviculture and pastures dedicated to livestock). We collected a total of 1266 dung beetles distributed in 35 species and classified into 11 functional groups. The taxonomic and functional diversity analyses showed that habitats that still maintain an arboreal stratum do not present differences between them, in contrast to habitats dedicated to livestock where there was a significant loss of species and functional groups. The distance between the trees, as well as the air and soil temperatures were determining factors in the selection of species and functional groups. Some of these environmental factors explain the differences in functional traits, represented as varying abundances of the species found. The rates of manure removal from the ecosystem were positively correlated to taxonomic and functional richness as well as biomass of beetles. Thus, we can conclude that habitats with tree strata have the capacity to preserve a larger proportion of the regional set of species as well as the important ones, while preserving the taxonomic and functional diversity and the ecosystem functions, such as the excrement removal rate.

## Introduction

One of the greatest global environmental problems is the loss of biodiversity as a consequence of human activities, mainly caused by habitat destruction [1, 2]. Landscape transformation and intensification of monocultures have led to changes in ecosystems, such as changes in plant community structure and loss of diversity, as agricultural or livestock substitute more diverse and heterogeneous natural ecosystems for homogeneous and simple ecosystems [3, 4]. These activities have led to the extinction of populations and species, without being able to assess, in most cases, their impact on functioning of biological systems [5]. Thus, there is an urgent need to quantify and predict the effects of disturbance on biodiversity patterns to guide conservation and management efforts of natural resources [6].

Studies on diversity patterns attempt to unravel how species behave in natural communities and under different degrees of disturbance. This has helped to explain the behavior of biological diversity and to what extent human actions can transform its dynamics, structure, and behavior [7]. Richness and abundance, along with diversity indexes, are the most commonly used measurements to assess impact on communities, but they treat all species in the same way in their contribution to the functioning of ecosystems [5]. These measures, while allowing for assessments of changes in patterns of diversity, are limited to describing which species are lost and how the loss of certain life forms could alter the structure and functioning of ecosystems [6].

Using measurements of various morphological, physiological, and life history traits that affect the biological performances of individuals (functional traits), measurements can be made that describe the different life strategies of the species in order to predict the response the organism will have to strong environmental changes. Thus, we would be able to infer its possible impact on the structuring of communities and ecosystem processes [8, 9]. Moreover, it is proposed that the functional component of biological diversity may be the key to understanding the mechanisms of community assembly and ecosystem processes, as well as the services they provide [8, 10, 11]. Therefore, functional diversity offers a mechanism to approximate the causal relationships existing between factors that promote local and global environmental changes, biodiversity, ecological functioning, and ecosystem services [12]. More diverse communities could have more ecosystem functions that could increase productivity (higher ecosystem services). In addition, differences in functional traits between organisms increases the total capture of resources. Both the identity and diversity of organisms together control the functioning of ecosystems [6]. Although the functional diversity affects the integrity of ecological processes and ecosystem dynamics, there is no simple and direct way of evaluating it [5, 13, 14]. Nevertheless, functional diversity can be quantified as the number of trophic levels or functional groups, as well as the resources used by the species or by using multivariate methods that summarize the functional variability in the group of species analyzed.

Functional diversity measurements have been used in many ways over the past two decades, with continuous advances of multivariate measurements [8, 15]. Villeger et al. (2008) [16] proposed three complementary and independent indices to assess the main facets of functional diversity: functional richness, functional equitability, and functional divergence [8, 17]. However, despite the wide use and sensitivity of these indices to environmental changes, these are still difficult to interpret biologically and difficult to compare with taxonomic diversity measurements. On the other hand, the diversity indexes proposed by Chao et al. (2014) [18], based on Hill numbers, permits the measurement of diversity from the effective number of species in the case of taxonomic diversity, or in the case of functional, it can measure the effective number of functional groups, which could be easily identified. This type of diversity measurement makes it easier to compare data from different communities because of standardization based on sample size. Moreover, it provides a unified framework to measure diversity between taxonomic, phylogenetic, and functional diversity [18].

Our study is focused on the diversity of dung beetles, which have been used as indicators since they have a great potential to respond to environmental changes [19–24]. They are also known for their role in ecosystem functioning due to their dependence on the excrement of vertebrates, particularly mammals, as a food source and as a nesting ground during reproduction [25–27]. The activities of these beetles are linked to a wide variety of ecological processes, including decomposition and displacement of excrement, secondary seed dispersal, incorporation of organic matter in the soil, bioturbation (displacement and mixing of soil particles), and control of flies and other parasites that affect cattle, pets, and humans [28]. Since nutrient cycling and soil formation are ecosystem functions that are strongly associated with the functioning of ecosystems, they are considered as supporting ecosystem functions, and are the basis of other functions. Thus, the conservation of the biota responsible for such processes is fundamental in maintaining ecosystems, and consequently, human well-being [29, 30].

The vegetation structure and the spatial and temporal availability of the excrement in a given habitat affects the community structure of dung beetles. The richness and equitability of communities may decline significantly among forest plantations and pastures and clearings, compared to mature forests or forests in advanced succession [22, 31–36]. Historical and/or ecological factors may have acted in the past or present as casual agents of loss or increase in diversity [37, 38]. Thus, elucidating which environmental factors have a greater influence on community structure facilitates the understanding of the relationships between environmental factors, diversity measurements, and functional traits [39, 40].

Although habitat transformation causes species loss, information on the consequences of such changes on functional diversity and ecosystem functions is still being studied. The loss of some functional groups of dung beetles, such as large excavator species, has a greater effect on the performance of ecological functions [41, 42], such as excrement removal and secondary seed dispersal compared to smaller and less efficient species for this type of function [5, 30, 41, 43]. Nonetheless, the effect of certain groups is considerable on ecosystem functions.

Since landscape transformation causes changes in the dung beetles’ diversity, those changes affect an important function of the ecosystem such as the cycling of nutrients. The objective of this work was to identify and evaluate the effect that different environmental factors of four habitats (with different degrees of disturbance) produce on the dung beetles’ taxonomic and functional diversity. In addition to this, we intend to evaluate the impact of diversity variation (taxonomic and functional) over the rates of excrement removal. Our hypothesis is that changes in environmental factors lead to alterations in the diversity of dung beetles (Scarabaeinae), thus damaging the ecosystem functions.

## Material and methods

### Study area

The study was conducted in the municipalities of Bom Retiro and Rancho Queimado in the state of Santa Catarina, southern Brazil. The study area belongs to the Atlantic Forest ecoregion and is located at an altitude between 800 and 1000 m.a.s.l. The area has a subtropical humid climate (Cfa) according to the Köppen-Geiger classification, with defined seasons and well distributed rain throughout the year, with an average annual rainfall of 1,700 mm. Temperatures vary strongly throughout the year, between 0°C and 40°C, with an annual average of 19°C [44].

Sampling was done at 15 sites located throughout four habitats: Mature forests (MAF) (4 sites), Early succession forests (ESF) (4 sites), Pinus monoculture (PIN) (3 sites) and open pastures for livestock (PAS) (4 sites). The sampling sites were located in four different areas, two in Bom Retiro (Area 1: 2753’41.71”S, 4925’57.61”O and Area 2: 2754’13.67”S, 4925’57.42”O) and two in Rancho Queimado (Area 3: 2740’24.61”S, 4902’63”O and Area 4: 2741’18.49”S, 4900’56.32”O). These sites are on private land and their owners have given permission to conduct the study and no specific permissions were required for the activities. All habitats were represented in each location, except for the Area 3 which did not have Pinus habitats (PIN) at all. The habitats within each area are contiguous, with the sampling sites located at a minimum distance of 200 m between them. The distance between areas (areas 1 and 2) in the municipality of Bom Retiro is 1 km; and in the municipality of Rancho Queimado it is 2.5 km (areas 3 and 4); and between both municipalities the distance is 46 km.

### Sampling of copro-necrophagous beetles

At each sampling site, we established five collection points distributed along a transect and distanced from each other by 30 m. At each collection point, two pitfall traps were installed with baits to collect the beetles, 15 m apart between pairs, one trap was baited with human feces (20g) and one with decomposing pork (20g) to attract coprophagous and necrophagous species respectively, totaling 10 traps per sampling site. The pitfall traps consist of a plastic container (20 cm in diameter, 20 cm in depth), placed on the ground level, filled halfway with a mixture of water and liquid detergent. This method is the most commonly used to collect copro-necrophagous beetles and the most effective to collect most species of this group. This protocol was replicated twice for each sampling site over four months between November 2017 and February 2018 in order to obtain a representative sample of the dung beetles community. The traps were left open for 48 hours and were examined every 12 hours (daytime and night-time) until the whole exposure was completed. The beetles were captured and preserved in a 90% alcohol solution. Later they were counted and identified at the species level, and the identification was confirmed by Dr. Fernando Vaz-de-Mello. The collected material was deposited in the Entomological Collection Mitia Heusi Silveira of the Center of Biological Sciences at the Federal University of Santa Catarina and in the Entomological Collection of the Federal University of Mato Grosso. This study did not involve endangered or protected species.

### Excrement removal

To evaluate excrement removal, we conducted an experiment at each sampling site, which consisted in depositing four samples of 85 g of fresh dog feces, spaced 50 m apart and placed above a 10 cm^2^ plastic mesh. After 48 hour of exposure the samples were weighed in order to measure the percentage of removal by coprophagous beetles. Feces were previously collected in the *Biotério Central* of the Federal University of Santa Catarina and frozen until the moment of use. Dog feces were chosen for this experiment because of their large availability in a controlled environment and also due to their ability to attract coprofauna in natural environments which is a key feature of omnivore species’ feces [45]. Additionally, dog feces have the same or even a better quality (food and brood balls’ number/weight) than those of the wild species [46]. In addition to the samples to measuring removal, we also placed two feces samples of the same weight protected by a voile screen, which avoided beetles’ reaching the feces, in order to measure the loss of weight by desiccation or percolation.

### Environmental data collection

At each sampling site, we measured different environmental and soil factors, adapting different methodological proposals [24, 47, 48]. Air and soil temperature were evaluated every 15 minutes; during the collection days by HOBO pro dataloggers. From the set of data obtained, we calculated the average daily temperature at each sampling site.

The vegetation structure for each sampling site was described by 10 environmental variables using the point-centered quarter method [47]. Two points were selected for sampling the vegetation structure, at each point, with the aid of a compass, a cross was drawn in a north-south and east-west direction, in order to locate the quadrants for evaluation. The following variables for trees, shrubs, and soil were measured in each quadrant: (1) diameter at breast height [DBH] of tress with a DBH > 5 cm; (2) tree height; (3) distance of tree to the center of the quadrant; (4–6) repetition of previous measurements for shrubs with a diameter at ankle height [DAH] ≤ 5 cm and minimum height of 1 m. In an area of one square meter within each quadrant the following variables were visually evaluated: (7) percentage of litter; (8) green cover; (9) bare soil; and (10) and depth of litter, measured in the center of the quadrant with the aid of a millimeter ruler.

Soils were evaluated with unmodified and composite samples. Unmodified samples were used to measure bulk density through the volumetric ring methods, which consists of taking a sample of the surface soil with the aid of a steel ring of known volume, drying the soil in an oven at a temperature of 105°C for 48 hours, and then weighing the soil to find the weight to volume ratio. The collection of composite samples consisted of obtaining five samples (one at each collection point) from the superficial soil, in a 10 x 10 cm area that is 15 cm deep. Afterwards the samples were mixed in a canvas bag and a 500 g portion was removed and subsequently analyzed according to *Embrapa* protocol to measure grain size (texture) and moisture percentage [48].

### Functional traits of species

To evaluate the functional traits of the species collected, we gathered a random sample of 15 individuals of each species. Subsequently, we pondered several characteristics of the specimens that may influence directly their fitness, including both morphological and behavioral characteristics from quantitative or qualitative nature. Qualitative data were associated with categorical variables, which were allocation of the resource: paracoprid, telecoprid or endocoprid; time of activity: diurnal or nocturnal, and diet: coprophagous, necrophagous and generalist. The quantitative measurements were: size: defined by biomass; flight ability: inferred by the ratio of wing length x wing width over the length of the body; wind shape: evaluated by the ratio of wing length to wind width; excavation capacity: inferred from the ratio of the anterior tibia to the length of the body; ability to roll: inferred from the ratio between the posterior tibia and body length; muscle strength: inferred by the size of the thorax (height of the beetle at the base level of the elytra x elytra width over body length). Length measurements were taken with a calliper and weight measurements were taken with a laboratory balance.

### Data analysis

The dung beetles communities from habitats were compared using the confidence intervals (95%) (Chao1 estimator) of the rarefaction curves and extrapolation of Hill numbers of either the richness (q = 0) or the diversity values (q = 1 and q = 2) [18]. This analysis was done separately for each area to have a clearer view of the behavior of the four communities from each locality [18]. In order to test the differences in taxonomic richness between habitats we used a Generalized Linear Mixed Model (GLMM) (Std. Error = 2.12), with the area as a random variable, in order to exclude the effect of differences in richness that are present in the four study areas.

We verified the differentiation of the communities according to the habitats with different land uses through a multivariate analysis of variances (Permanova), using the function *adonis*, with 999 permutations in the *vegan* package [49]. In order to visualize the results we applied a principal coordinate analysis (PCoA) using the Bray-Curtis dissimilarity.

Based on the matrix of functional traits, we calculated the dissimilarity between species using the Gower index. This index is useful when the variables used are a combination of numeric and non-numeric variables (nominal, binary, ordinary, even combinations between them). From the Gower index, a dendrogram was created to explore the differences between functional groups. To make these groups we used the hierarchical grouping method through partitioning with *divisive* technique (function *diana* of the *cluster* package). This method starts by considering the complete data set as a cluster and then splits the groups until each object is separated [50]. The divisive methods have the advantage of considering many divisions in the first step, reducing the probability of a wrong decision, thus, it is more secure than the agglomerative hierarchical methods [48].

The functional groups obtained were included in the functional diversity analysis. Similarly to the taxonomic diversity, the functional diversity were estimated and compared using the confidence intervals (95%) (Chao1 estimator) of the rarefaction curves and extrapolation of Hill of the richness (q = 0) and diversity values (q = 1 and q = 2).Thus, this method unifies the comparison method of taxonomic and functional diversity, as proposed by Chao et al. (2014). To compare functional richness among habitats, we repeated the same method we used in the taxonomic analysis (GLMM) (Std. Error = 0.94), including areas as random variables to exclude the effect of the differences in richness that are present in the four study areas.

To explore the relationships between taxonomic and functional diversities with environmental factors, we evaluated the values of the taxonomic and functional richness from the 15 sample sites as a response variable and the environmental variables of the same 15 sites as explanatory factors, in order to understand which of these variables operates as a limiting factor and species selector in the community. Therefore, we used generalized linear models (GLM) with negative binomial distributions [51, 52]. Afterwards, GLMs were used to explore the relationship between the excrement removal rate as a response variable and the community measurements as explanatory variables (taxonomic and functional richness (q = 0), taxonomic and functional diversity (q = 1 and q = 2), abundance, and biomass). Variables that presented high correlation were excluded from the model.

We used a “fourth corner” model to understand how different functional traits relate to environmental factors, which models species abundance according to environmental variables (by habitat), species traits (by species), and the interaction between them. In the fourth corner analysis, the relative abundance of the species from a sample site is expected to differ from the regional relative abundance due to differences in functional traits, such that the increase or decrease in abundance of a species is related to the presence of better or worse species traits that are suitable for the environmental variables in a particular sampling site. On the other hand, the fourth corner models are based on generalized linear models (GLM), and are able to control the strong mean-variance relationship in abundance data instead of using and transforming methods that assume equal variance, which generates advantages in interpretation, verification of models, extensions, and inferences [51]. The GLMs used in the fourth corner model were adjusted using the LASSO penalty (*mvabund* package). This simplifies the interpretation because it automatically performs model selection, setting to zero any interaction coefficient that does not reduce the Bayesian Information Criteria (BIC) [53].

All analyzes were performed using R software version 3.4.3 [54].

## Results

### Taxonomic diversity

A total of 1266 dung beetles were collected from 35 species (S1 Appendix). From the total species captured, 24 were collected in the Mature forests (MAF), 28 in the Early succession forests (ESF), 24 in the Pinus monoculture (PIN) and 13 in the pastures (PAS). We generally observed that pastures habitats showed a marked decrease in species and individuals compared to forest habitats (MAF, ESF) and Pinus monocultures, which maintained similar richness, although with slightly lower values in the MAF habitats (Table 1).

**Table 1:** Richness and abundance in four study areas in the municipalities of Bom Retiro (area 1 and 2) and Rancho Queimado (area 3 and 4), in the state of Santa Catarina, Brazil.

	Area 1				Area 2				Area 3			Area 4			
	MAF	ESF	PIN	PAS	MAF	ESF	PIN	PAS	MAF	ESF	PAS	MAF	ESF	PIN	PAS
**Species**	7	8	8	2	11	11	11	3	14	18	2	14	20	18	8
**Abundance**	121	65	61	13	50	71	73	3	123	124	2	109	163	249	39
**Species—Area**	13				17				20			25			
**Abundance—Area**	259				197				249			561			

Mature forests (MAF), Early succession forest (ESF), Pinus monoculture (PIN) and Pastures (PAS).

**Fig 1:**
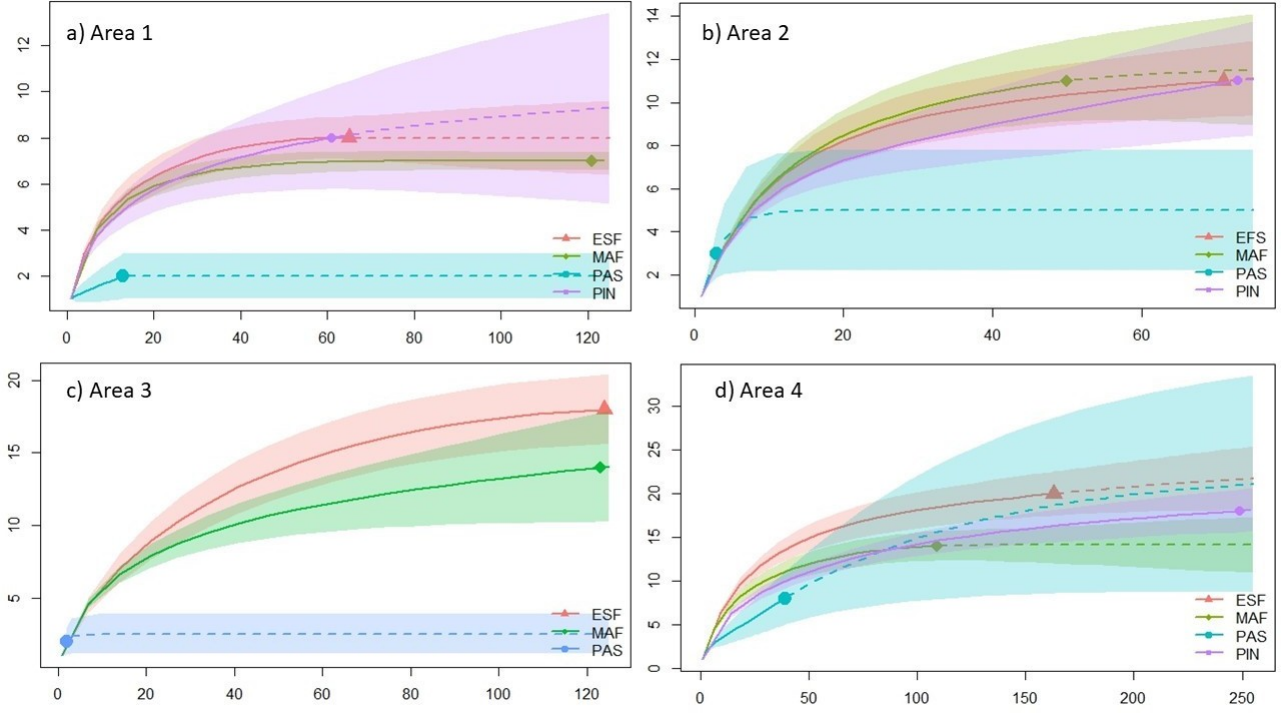
Rarefaction and extrapolation with Hill Numbers (q = 0) (y-axis = Species diversity (q0); x-axis = abundance) for species taxonomic richness in Mature forests (MAF), Early succession forests (ESF), Pinus monoculture (PIN) and Pastures (PAS) (a) Area 1 in Bom Retiro, SC (b) Area 2 in Bom Retiro, SC (c) Area 3 in Rancho Queimado, SC (d) Area 4 in Rancho Queimado, SC, southern Brazil.

Sampling coverage indicated sample adequacy at most of the sample sites, with values above 95% of the estimated total; the only exception was the pastures habitats in areas 2, 3, and 4, which presented values of 35%, 65%, and 85% respectively (Table 2). The rarefaction curves showed that there was no significant difference (superimposed confidence intervals) in taxonomic richness among forest habitats (MAF, ESF, PIN). Whereas, the pastures habitats presented smaller values, showing a marked decrease in richness, preserving only around 18% of the species (Fig 1). In contrast, area 4 showed a high richness in the pastures and no significant differences in richness estimated compared to the other habitats (Fig 1D).

**Table 2:** Taxonomic diversity based on Hill numbers.

	Area 1				Area 2				Area 3			Area 4			
	MAF	ESF	PIN	PAS	MAF	ESF	PIN	PAS	MAF	ESF	PAS	MAF	ESF	PIN	PAS
**Estimate richness (q = 0)**	7	8	9.2	2	11.5	11.1	11.1	5	14.1	18	2.5	14.1	20.6	16.9	19.9
**CI**	6.7–7.2	6.5–9.4	6.2–12.1	1.0–2.9	8.6–14.3	9.4–12.7	7.6–14.6	2.3–7.6	10.4–17.6	15.6–20.3	1.1–3.8	11.1–17.1	17.3–23.8	14.8–19.2	8.5–30.6
**Diversity (q = 1)**	5.4	5.8	5.1	1.4	8.8	8.2	7.4	8.4	8	9	-	9.6	12.9	7.7	4.6
**CI**	4.7–6.0	4.7–7.0	3.9–6.2	0.9–1.8	7.0–10.6	7.1–9.2	6.0–8.8	2.9–13.8	6.8–9.1	7.2–10.9	-	7.8–11.5	11.6–14.3	6.5–8.9	2.2–7.0
**Diversity (q = 2)**	4.5	4.7	3.9	1.1	7.2	7.2	6.3	75	6.1	5	125	7.3	9.6	5.3	2.7
**CI**	3.8–5.2	3.4–5.9	3.0–4.7	0.6–1.7	5.3–9.1	6.0–8.5	5.3–7.3	7.1–142.8	4.8–7.3	3.5–6.4	2.3–247	5.9–8.8	7.8–11.3	4.4–6.1	1.9–3.5
**Sample coverage (%)**	100	100	90	100	95	100	95	35	95	95	65	100	100	100	85

Richness (q = 0), diversity (Shannon, q = 1 and Simpson q = 2) and sampling coverage (for q = 0) in four areas in Santa Catarina State, south of Brazil, in Mature forests (MAF), Early succession forests (ESF), Pinus monoculture (PIN), Pastures (PAS).

The loss of richness was confirmed for pastures in comparison to early succession forest (F = 6.82; P < 0.001), mature forests (F = 5.03; P < 0.001), and Pinus monoculture (F = 5.32; P < 0.001). (S2 Appendix).

The environmental variables explained the decrease of the taxonomic richness in the pastures (Table 3). The GLM result showed that the set of explanatory variables explains 90% of the variable response of the model. The distance between trees (Z = -5.89; P < 0.001), which was an inverse indicator to density, and soil temperature (Z = 4.19; P < 0.001) were the main parameters associated with taxonomic richness in copro-necrophagous beetles. Thus, the model showed that as tree density decreased and temperature increased, species richness decreased; this pattern was mainly observed in pastures.

**Table 3:** Statistics of the Generalized Linear Model (GLM) used to assess the relationship between the explanatory variables [DBH, tree height, distance between trees, DAH, shrub height, distance between shrubs, percentage of litter; green cover, bare soil, depth of litter, clay, sand, lime, moisture] and the taxonomic richness.

	Estimate	Std. Error	z value	Pr(>|z|)
(Intercept)	-1.14E+01	3.71E+00	-3.068	0.00216
Dist tree	-2.80E-03	4.75E-04	-5.893	3.79E-09
% litter	7.33E-03	4.77E-03	1.537	0.12424
Soil Temp	8.26E-01	1.97E-01	4.193	2.75E-05
Null deviance: 48.0748 on 14 degrees of freedom				
Residual deviance: 4.5529 on 11 degrees of freedom				
AIC: 76.21				

Diversity analyzes, including relative abundance of species, had a similar result to richness analyzes, where confidence intervals of estimated diversity were superimposed between forest and Pinus monoculture habitats, for both q = 1 (Shannon) and q = 2 (Simpson) (Table 2). In regards to communities from pastures habitats, there was significantly lower diversity values than those from the forested areas (MAF, ESF, PIN); however, some diversity estimators within pastures habitats had values that were not very reliable in relation to the number of typical or dominant species, due to the few individuals found in these habitats (Table 2). Nevertheless, areas with canopy presence (MAF, ESF, PIN) have high diversity, since the typical species (Shannon, q = 1) can represent between 50% and 70% of the total species per habitat, and abundant species (Simpson, q = 2) represent between 30% and 50% of the species richness values, which result from the high equitability in the abundance of species in these communities (Table 2). In terms of the similarity among the communities from the taxonomic composition of species and their abundance (Bray-Curtis dissimilarity), we found that there was a significant difference among the four habitats (F = 1.398; P = 0.003). Habitats which have conserved their canopy maintained a similar structure of dung beetle communities among them, but it was significantly different in pasture habitats where there were fewer dung beetle communities.

### Functional diversity

#### Building functional groups

**Fig 2:**
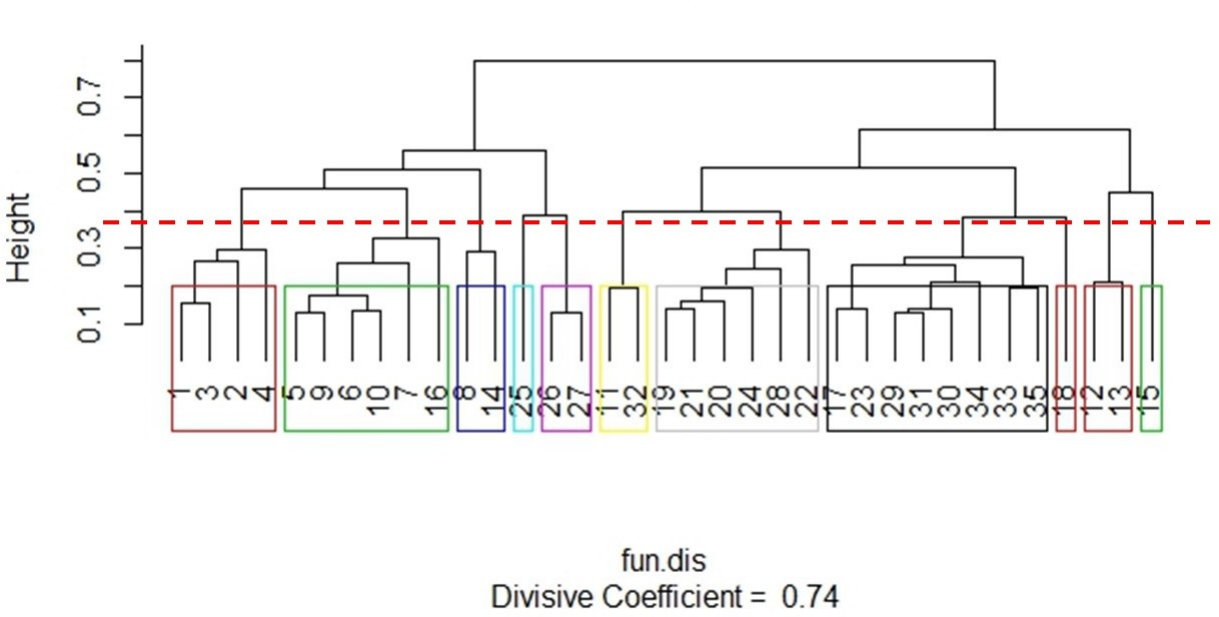
Dendrogram showing the 11 functional groups produced from the Gower index with divisive hierarchical clustering technique with the functional attributes of 35 species of scarab beetles. Functional groups were considered at an arbitrary Gower distance (dotted line).

Based on the functional traits of the 35 species (S4 Appendix), 11 groups were created from the grouping generated with the Gower Index (Coefficient = 0.74) (Fig 2). The groups were characterized as follows:

- Group 1 (Red): Small, paracoprids, diurnal, and generalist species. The following species can be found in this group: *Canthidium* aff *dispar* (1), *Canthidium* aff *sulcatum* (2), *Canthidium* aff *trinodosum* (3), and *Canthidium* sp2 (4).
- Group 2 (Green): Medium, telecoprids, diurnal, and a coprophagous or generalist species. Species: *Canthon* aff *mutabilis* (5), *Canthon angularis* (6), *Canthon lividus* (7), *Canthon Oliveroi* (9), *Canthon rutilans* (10), and *Deltochilum rubripenne* (16).
- Group 3 (Blue): Medium, telecoprids, nocturnal, and a preferentially necrophagous species. Species: *Canthon luctuosus* (8) and *Deltochilum morbillosum* (14).
- Group 4 (Aquamarine blue): In this group there is only one species–*Eurysternus cyanescens* (25), which is medium, endocoprid, nocturnal, and has a generalist species.
- Group 5 (Purple): Medium, endocoprids, diurnal, and a coprophagous species. Species: *Eurysternus inflexus* (26) and *Eurysternus parallelus* (27).
- Group 6 (Yellow): Large, paracoprids, diurnal, and a coprophogaus or generalist species. Species: *Coprophanaeus saphirinus* (11) and *Phanaeus splendidulus* (22).
- Group 7 (Grey): Large, paracoprids, nocturnal, and coprophagous species. Species: *Dichotomius assifer* (19), *Dichotomius fimbriatus* (20), *Dichotomius fissus* (21), *Dichotomius Mormon* (22), *Dichotomius sericeus* (24), and *Homocopris* sp (28).
- Group 8 (Black): Small and medium, paracoprids, nocturnal, and a coprophagous species. Species: *Dichotomius* aff *acuticornis* (17), *Dichotomius opalescens* (23), *Onthophagus* aff. *Hirculus* (29), *Onthophagus tristis* (31), *Onthophagus catharinensis* (30), *Uroxys dilaticollis* (33), *Uroxys* sp. 1 (34), and *Uroxys* sp. 2 (35).
- Group 9 (Red): In this group there is only one species–*Dichotomius Ascanius* (18), medium, paracoprid, nocturnal, and is different from the previous group because it has a generalist species.
- Group 10 (Red): Very large, telecoprids, nocturnal, and coprophagous species. Species: *Deltochilum brasiliensis* (12) and *Deltochilum dentipes* (13).
- Group 11 (Green): In this group there is only one species -*Deltochilum multicolor* (15), which is large, but smaller than the previous group, telecoprid, nocturnal, and a preferentially coprophagous species.

#### Functional diversity analysis

Functional richness was greater in forest environments, since it varied between six and 10 groups in comparison to the pastures areas, which only had one or two functional groups (except for area 4, which did not have a significant different to other sample sites in the same area) (S5 Appendix). Group 1, which contained small and diurnal paracoprids species, and Group 6, which contained large and diurnal paracoprid species, were found in all the forest habitats and Pinus monocultures, but absent in pastures areas. A similar pattern occurred with group 2, which contained medium telecoprid species, and group 10, which contained large nocturnal telecoprids species, that are well represented in the areas with tree cover, but few individuals are found in open areas. Groups 4 and 5 (endocoprid species) and 9 (medium, nocturnal and paracoprid species) were only found in forested areas (MAF, ESF) or in Pinus monoculture areas (PIN), although not as frequent as the groups 1 and 6. Group 7, large nocturnal excavator species, and group 11, which contained one large nocturnal roller species, are groups whose abundance is considerably affected by the absence of a canopy, even though they were found in pastures.

In general, pastures showed a marked decrease of functional richness, preserving only a small set (22%) of the functional groups found in forest habitats (MAF, ESF, PIN). The species found in the pasture habitat usually includes small species (group 2 and group 8) and in particular cases some individuals from groups 7 and 10.

The sampling coverage conducted using the functional groups formed from the functional trait grouping analysis showed sample adequacy in most habitats, with values above 90% of the estimated total, except in the area 3 pastures habitat, which had a value of 65% of sample coverage (Table 4).

**Table 4:** Functional diversity based on Hill numbers.

	Area 1				Area 2				Area 3			Area 4			
	MAF	ESF	PIN	PAS	MAF	ESF	PIN	PAS	MAF	ESF	PAS	MAF	ESF	PIN	PAS
Estimate richness (q = 0)	6.0	6.0	6.0	2.0	7.3	8.0	5.0	1.0	10.0	10.0	2.5	8.0	10.0	9.0	10.0
CI	6.0–6.0	5.3–6.6	5.0–7.0	1.0–2.9	5.1–9.5	7.0–9.0	5.0–5.0	1.0–1.0	8.6–11.4	9.0–11.0	1.2–3.7	6.7–9.2	9.0–11.0	8.5–9.4	9.0–11.0
Diversity (q = 1)	4.2	4.5	4.5	1.4	5.6	5.6	4.0	1	5.8	5.8	NA	5.7	7.4	5.7	3.5
CI	3.6–4.8	3.8–5.2	3.6–5.3	0.8–1.9	4.4–6.6	4.5–6.6	3.5–4.5	1.0–1.0	4.8–6.8	4.7–6.8	NA	5.0–6.5	6.6–8.1	5.2–6.3	2.3–4.7
Diversity (q = 2)	3.5	3.8	3.6	1.1	4.9	4.3	3.6	1	4.4	4.1	125	4.9	6.2	4.7	2.5
CI	3.0–4.0	2.9–4.7	2.8–4.5	0.8–1.5	3.7–6.0	3.1–5.4	3.0–4.1	1.0–1.0	3.6–5.1	3.3–4.9	2.2–247	4.2–5.6	5.3–7.2	4.1–5.2	1.8–3.2
Sample coverage (%)	100	100	100	100	100	100	100	100	100	100	65	100	100	90	100

Richness (q = 0), diversity (Shannon, q = 1 and Simpson q = 2) and sampling coverage (for q = 0) in four areas in Santa Catarina State, south of Brazil, in Mature forests (MAF), Early succession forests (ESF), Pinus monoculture (PIN), Pastures (PAS).

The functional richness analysis used the same method as that used for the taxonomic richness (q = 0 from Hill numbers), showed that the functional richness in forest habitats (MAF, ESF, PIN) was greater than in pastures habitats (Table 4). As to taxonomic richness, functional richness showed a similar tendency, where the areas with canopy presence did not have a significant difference between them (rarefaction curves with overlapping confidence intervals), but there was a loss of functional groups in the pasture habitats. This trend was only different in area 2 (Table 4), where Pinus habitat and forests in advanced succession did not overlap in rarefaction curves. In area 4, as there was also the case for taxonomic richness, due to the presence of sparse trees in the pastures environment, there were no significant differences in the functional richness estimated by habitat (Table 4).

Differences in functional richness considering the area as a random variable showed a significant decrease between pastures habitats compared to forest habitats in early succession (F = 5.84, P <0.001), forests in advanced succession (F = 5.56; P <0.001) and Pinus monoculture (F = 4.09; P <0.001). The environmental variables also explained the decrease in functional richness in the pastures within different areas. The distance between the trees (Z = -4.458; P <0.001) best described the loss of functional richness of copro-necrophagous beetles in open areas, and to a lesser extent environmental temperature (Z = 1.87; P = 0.06), with a percentage explanation of 85% of the variation. The GLM model revealed again that as the density of trees decreases and the temperature increases (this time the air temperature and not the soil as in the analysis of the taxonomic richness), the functional richness decreases (Table 5).

**Table 5:** Statistics of the Generalized Linear Model (GLM) used to assess the relationship between the explanatory variables [DBH, tree height, distance between trees, DAH, shrub height, distance between shrubs, percentage of litter; green cover, bare soil, depth of litter, clay, sand, lime, moisture] and the functional richness.

	Estimate	Std. Error	z value	Pr(>|z|)
(Intercept)	-2.13E+00	2.82E+00	-0.755	0.45
Dist tree	-1.87E-03	4.64E-04	-4.034	5.48E-05
Air Temp	3.06E-01	1.63E-01	1.877	6.06E-02
Null deviance: 23.2076 on 14 degrees of freedom				
Residual deviance: 3.4975 on 12 degrees of freedom				
AIC: 65.792				

The functional diversity (q = 1 and q = 2 from Hill numbers) had a high equitability among the functional groups present in the MAF, ESF, and Pinus areas, and consequently, had a high diversity, with no significant differences between them for Shannon and Simpson (Table 4). Similarly to taxonomic diversity, functional diversity analyses showed that between 50 and 70% of the present functional groups were typical (q = 1, Shannon) and between 30 and 50% were abundant (q = 2, Simpson). In contrast to forest and Pinus habitats, pastures habitats had significantly smaller diversities.

The functional composition of species showed a significant difference in the total composition of functional groups among the four habitats (F = 1.524; P = 0.013). Similar to taxonomic composition, the areas that conserve the arboreal stratum maintain a very similar functional composition in comparison to pastures habitats, where a separation of the composition of the forest and Pinus areas is observed.

**Fig 3:**
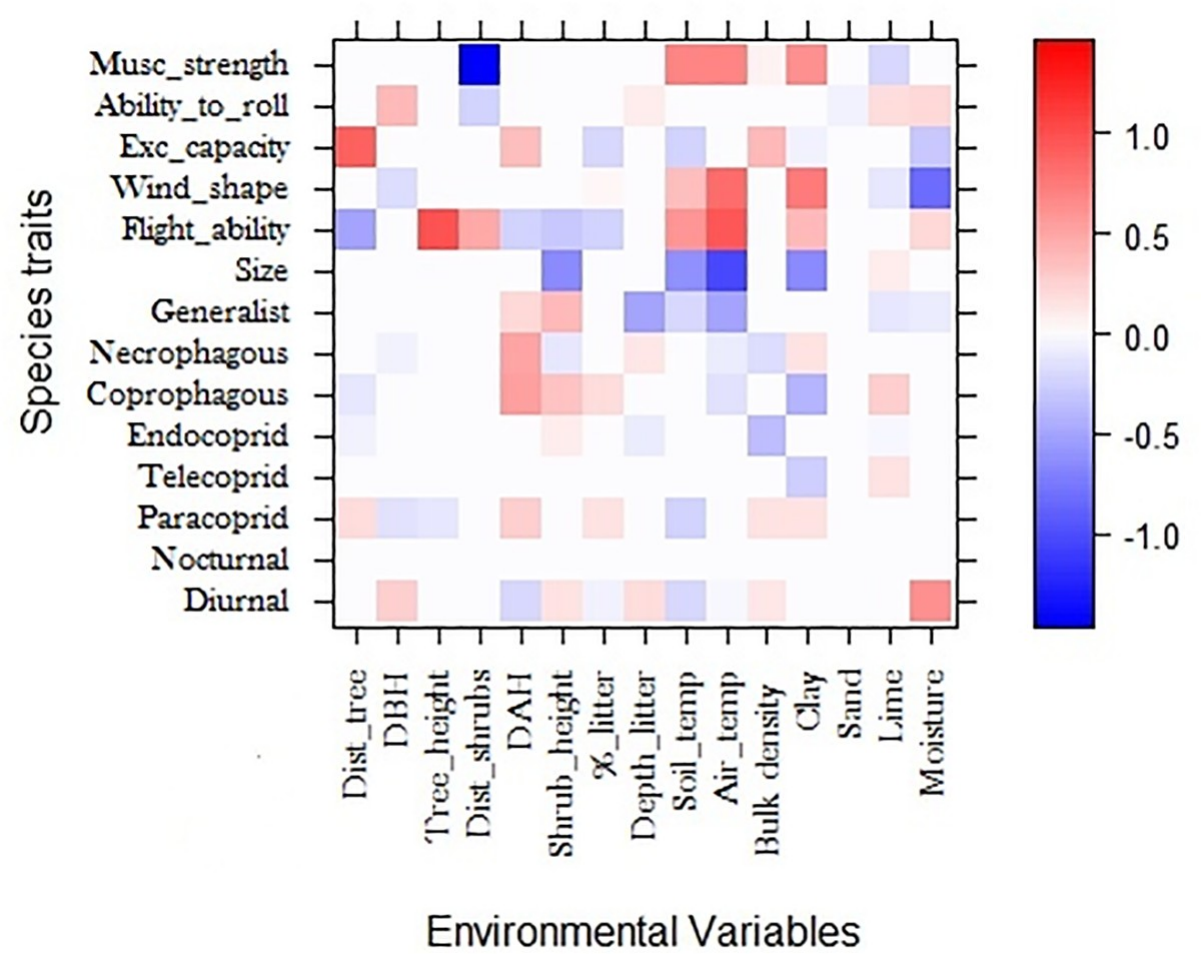
Results of the fourth-corner model in relation to traits-variable. Traits are colored according to their fourth-corner coefficients: red indicates a significant positive trait-variable association, and blue indicates a significant negative trait-variable association. Color depth indicates the strength of the trait-variable association.

According to the fourth corner analyzes, species traits and functional groups explain a significant amount of variation in the abundance distribution of species in relation to environmental variables. For species traits, each trait seems to be affected in a particular way by each environmental variable; however, we observed a general tendency with stronger (80–100%) and multiples relations to the variables distance between trees, percentage of clay, and soil and air temperature. Also, variables such as diameter and height of shrubs, percentage of soil moisture, and percentage of litter influenced multiple functional traits, although with less intense relationships (60–80%) (Fig 3).

In addition, the same analysis revealed that more robust species (Musc_strength) are associated with areas of low shrub density (80–100%), as well as high soil and air temperatures, and a large clay percentage (60–80%). The trait that relates the anterior tibia to the body width (Exc_capacity) was influenced by multiple environmental factors, where the factor distance between trees had a positive relationship, and was the factor that affected this trait more intensely (80–100%). Roller species (Ability_to_roll) had a positive relationship with DBH, litter depth, silt, and humidity, and had a negative relationship with distance between shrubs and percentage of sand, however, the positive and negative relationships were low intensity (5–15%).The trait associated with flight capacity (Flight_ability), measured as wing size in relation to the body, was related to multiple environmental factors. Larger and flying-skilled species were related to well-structured habitats The flight capacity trait had a negative relationship with pastures habitats (60–80%) and a positive relationship with trees height (80–100%). Factors associated with the presence of shrubs also influenced this trait (20–40%), such as the high air and soil temperatures, which had a positive relationship with this trait (70–90%). The wind shape had low intensity relationships, with a negative relationship to soil moisture (60–80%) and DBH (10–30%), and positive relationships with air (80–100%) and soil temperature (30–50%). Biomass had negative relationships with air (40–60%) and soil temperature (80–100%), as well as the shrub height and percentage of clay (40–60%).

Although traits associated with diet had less intense relationships (20–40%), they seemed to be related to variables such as the structure of shrubs, presence of litter, temperature and soil density. The paracoprid behavior (Paracoprid), however, had weak relationships (10–20%), appearing to be related to multiple environmental variables, such as distance between trees, DBH, tree height, air temperature, and litter percentage. Additionally, diurnal behavior had weak relationships (10–20%) to habitats with high DBH values, presence of shrubs, litter depth, and moderate environmental temperatures, but with an intense relationship to moisture (40–60%).

### Ecosystem function: Excrement removal

**Fig 4:**
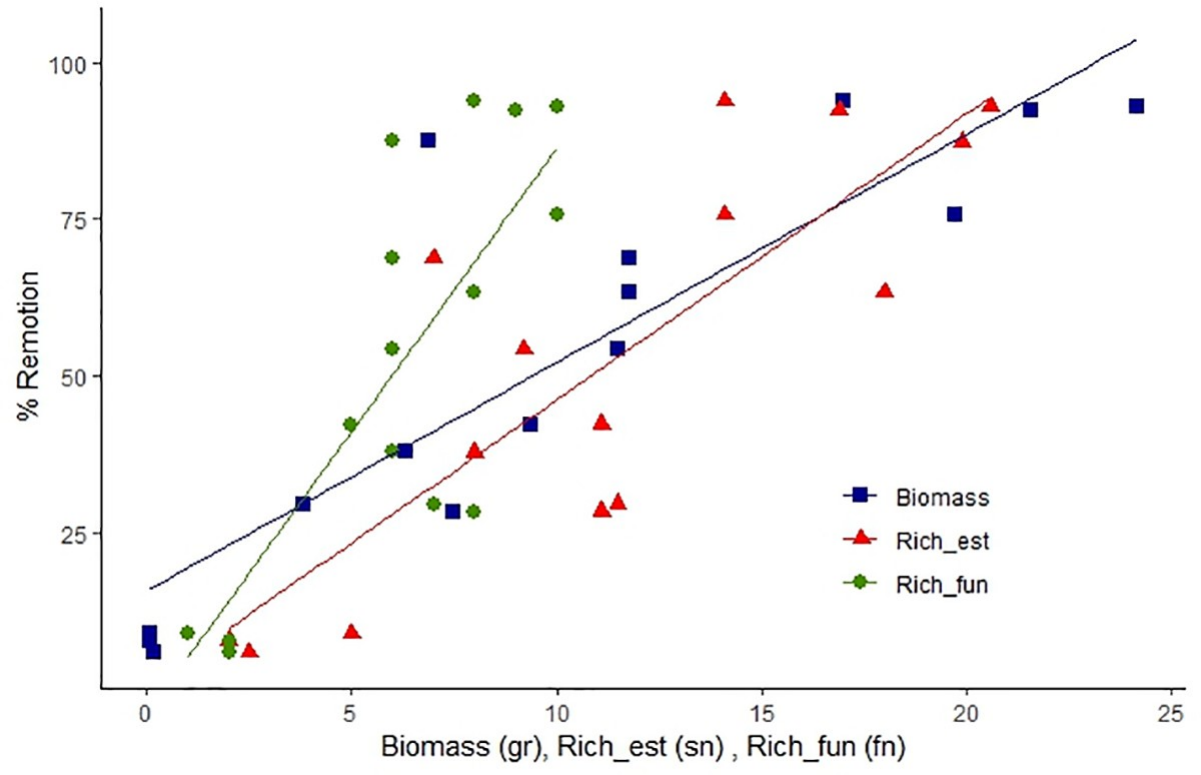
Relationship between biomass, estimated richness and functional richness in each studied habitat and excrement removal. Measurement units (sn: Species number; fn: Functional groups number; gr: Grams).

The removal of excrement by dung beetles was positively related to biomass (GLM, Z = 5.14, P <0.001) and estimated taxonomic richness (Z = 3.55, P <0.001) (Fig 4). Although functional richness was not significant in the model when all the community measurements were included, this variable is significant when taxonomic richness is excluded from the model; however, its high correlation with taxonomic variables causes functional richness to be excluded for presenting lower Z values (Z = 3.30, P <0.001). These analyzes show that loss of species per habitat, especially large species, negatively affects removal rates, with up to 90% of the removal capacity being lost in pasture areas dedicated to livestock.

The contribution of each functional group’s biomass showed that the presence of groups 6, 7 and 10 in the community of dung beetles, provided between 50 and 90% of the total biomass (Table 6). These results show that large species (diurnal and nocturnal), such as *Dichotomius*, *Phanaeus*, *Coprophanaeus* and *Deltochilum* beetles play a fundamental role in the removal function, since removal rates can decrease by up to 80% (Table 6) in the absence of these groups. Furthermore, these functional groups were associated with forested habitats which means that the ecosystem functions provided by dung beetles are lost on pasture habitats.

**Table 6:** Biomass contribution (g) of each functional group of dung beetles in four habitats with differences in land use: Mature forests (MAF), Early succession forests (ESF), Pinus monoculture (PIN), Pastures (PAS).

	Area 1				Area 2				Area 3			Area 4			
	MAF	ESF	PIN	PAS	MAF	ESF	PIN	PAS	MAF	ESF	PAS	MAF	ESF	PIN	PAS
Group 1	0.32	0.28	0.08		0.12	0.05	0.04		0.06	0.04		0.05	0.05	0.97	
Group 2	1.59	0.67	0.88	0.00	0.50	0.35	0.90		0.21	0.49	0.03	0.07	0.52	0.96	0.03
Group 3	0.08					0.01			2.18	0.86		1.17	0.39	0.47	0.08
Group 4					0.05				0.05	0.09					
Group 5						0.17			0.15	1.07		0.04	0.31	0.19	0.06
Group 6	2.39	3.76	1.71		1.71	3.08	2.39		6.50	1.03		5.83	6.38	8.24	
Group 7			0.36						8.43	5.67		6.04	8.80	8.14	3.64
Group 8	0.02	0.03	0.02	0.08	0.22	1.06	0.26	0.10	0.02	0.25		0.59	0.60	0.57	
Group 9		0.20			0.34	0.47							0.07		
Group 10	7.20	1.35	8.55		0.90	2.25	5.85		1.80	1.80		3.15	4.01	1.35	1.10
Group 11									0.15	0.44	0.15		2.94	0.59	1.91
Total biomass	11.76	6.32	11.49	0.08	3.83	7.48	9.38	0.09	19.72	11.77	0.18	16.95	24.17	21.59	6.88
Remotion (%)	68.67	37.82	54.32	7.79	29.62	28.39	42.28	8.89	75.66	63.33	5.83	93.91	92.95	92.38	87.39

## Discussion

We found that the landscape transformations caused changes in the diversity of copro-necrophagous beetles in habitats with different degrees of disturbance. The habitats with tree cover did not show differences in diversity among dung beetle communities, but there were significant changes in the pastures habitats dedicated to livestock. The excrement removal was strongly related to the richness and biomass of beetles, which were strongly influenced by tree density and air and soil temperatures.

Many studies have shown that areas that preserve part of the tree strata, including monoculture areas, have the capacity to maintain a large proportion of dung beetle fauna [32–34, 43, 55, 56]. In the case of our study area, we observed that a large part of the regional diversity has a high resilience with the capacity to colonize multiple habitats with different disturbance levels, as long as the tree cover is maintained, since a large number of dung beetles from the regional fauna are closely related to tree cover (ombrophilous species) [20].

This assertion is supported by the results obtained in this study, where distance between trees is the variable that best explains the richness of dung beetles. Habitats with tree cover have the capacity to shelter most of the taxonomic and functional richness available in the set of species. Even pastures with scattered trees had greater species richness in comparison to the other pastures habitats without trees, this was corroborated with the richness values in area 4, which had large trees (araucarias) in the pastures matrix (S3 Appendix), corroborating the model’s significance.

Evidently, the dung beetle community does not exhibit a gradual loss of species in relation to the degree of disturbance, but exhibits a substantial decline when the canopy is removed. This result can be explained by the similarity in environmental conditions (temperature, radiation, humidity), which could explain the taxonomic and functional similarity between the habitats, since habitats of Pinus and forests in early succession contain a subset of the beetle fauna present in more conserved environments [43].

Although the sampling coverage showed a sufficiency of more than 90% in most habitats, in the pastures habitats we observed percentages of taxonomic diversity between 35% to 85% (areas 2, 3 and 4) and 65% for functional diversity (area 3). Although the sampling coverage showed a sufficiency of more than 90% in most habitats, in the pastures habitats we observed percentages of taxonomic diversity between 35% to 75% (areas 2, 3 and 4) and 65% for functional diversity (area 3). Even though these results show a low sample effort, they were habitats that underwent the same sample treatment as other habitats in the study. These results are explained by the community disintegration in pastures, where up to 80% of copro-necrophagous beetles are lost. In this sense, there are so few individuals in these habitats that an individual captured (Singleton) at random from the sampling, affects the richness estimation, the sample coverage and the confidence interval. Not only is the loss of species corroborated by the values of richness and diversity, but also by the percentage of removal found in pasture habitats where there is a loss of up to 90% of the removal capacity due to the reduction of species performing this function.

The significant loss of species in pastures has already been observed in numerous studies comparing pastures to habitats with tree cover [20, 32, 35, 55]. As mentioned, the expansion and opening of new pastures is one of the disturbances with the greatest impact on beetle communities [19, 20]. This is because pastures are affected by abrupt microclimatic changes, raising the temperature and reducing humidity, resulting in a loss or replacement of species, which modifies the taxonomic and functional composition [43, 57, 58].

Our study suggests that most of the species present in the regional set of dung beetle species are not capable of colonizing to pasture habitats. This process is possibly associated with the natural history of the area, since it was originally covered with forests and had a low association with natural savannas, resulting in a reduced number of species adapted to survive and colonize pastures, which are currently transformed for livestock. An opposite case was found in the Brazilian Cerrado, where open areas or specifically areas of exotic pastures have the capacity to maintain a considerable proportion of both, richness and abundance of dung beetles associated with this type of transformed environment [59, 60]. Thus, the occupancy capacity of a given habitat will depend on the scenario that shaped the evolutionary history and generated the regional set of species. The species in our study area would have the capacity to withstand modifications in the environment as long as the tree canopy is conserved.

The variables distance between trees and soil temperature best explained the taxonomic richness. Absence of trees had a negative relationship with richness, which can be explained by the pastures allowing greater radiation input, besides having large temperature oscillations [61–63]. These oscillations cause more susceptible environments to select for species due to thermal variation, compared to environments with canopy presence, where there is lower radiation, lower temperature variation, offers greater stability, and less selection susceptibility. Thus, livestock represents a major impact on terrestrial habitats, as it modifies the composition of species and the dynamics of key ecological processes to the functioning of ecosystems, such as organic matter decomposition and nutrient cycling.

Ecosystem functions, specifically excreta removal, are closely linked to taxonomic richness and beetle biomass of each habitat, since a greater richness allows a greater number of guilds and/or functional groups. This implies a greater use of the resource. In addition to the multiplicity of niches, there is a tendency to maintain a greater number of large or larger biomass species with greater capacity for excrement removal [5, 30, 41, 43]. More diverse communities are more productive because they contain key species that have a greater influence on productivity, and differences in functional traits between organism’s increases the total resource capture.

The number of species that belong to the same functional group declines with the magnitude of the intervention, where pastures have a few taxa and functional groups, which is different to habitats with forest presence, where there is an increase in functional richness [5]. Additionally, greater functional diversity enhances certain ecosystem functions, such as removal. However, this does not mean that all species have the same role in ecosystem functioning, nor do they have the same responses to environmental changes [41, 64]. In other words, habitats with a greater variety of large species, even belonging to a single functional group, are habitats that tend to have higher values and greater stability of excrement removal rates [5, 41, 43, 64].

Presence of trees and temperatures were very influential variables on the functional traits evaluated, as well as other variables related to habitats with the presence of arboreal and shrub strata, such as DBH, DAH, and shrub height. The interaction between these traits and environmental variables were able to explain differences in species abundances. Flight capacity and biomass are traits related to the size of the species were negatively affected by the distance of trees and temperature, which means that they are strongly affected by deforestation or loss of tree cover. This phenomenon has already been reported by several authors [5, 41, 64] where large species, in addition to being more efficient, are also more susceptible to extinction.

On the other hand, the form of resource allocation was also affected by the distance of trees. The paracoprid species (especially the small species) had a greater advantage in open areas, since the paracoprid species can take advantage of the fresh interior of the manure, differing from the telecoprid species, which are more susceptible to superficial desiccation of the resource [42]. In relation to the temperature, we observed that the traits associated to flight are positively related, where larger species with greater flight capacity and species with more elongated wings are positively related; however, this phenomenon seems possible as long as tree cover is present, at least for large sized species.

## Conclusions

The present study demonstrated the strong impact that the open areas have on the diversity of dung beetles, as well as the ecosystem functions they provide. Areas without tree cover can lose up to 80% of the beetle community and up to 90% of the removal capacity in these environments. We found a high resilience of the community of dung beetles in forest environments with human intervention, as long as the tree cover is maintained. The ecosystem function of excrement removal was strongly related to the richness and biomass of beetles, which were strongly influenced by tree density and air and soil temperatures. The presence of trees was a key environmental variable associated to the diversity of dung beetles and their ecosystem functions. Finally, in terms of biodiversity conservation and its ecosystem functions, it is important to keep trees dispersed in the matrixes of open areas, as well as adjacent forests, facilitating the exchange of the dung beetle fauna between environments, and thus, maintaining the ecosystem services they provide.

## Supporting information

S1 Appendix Number of captured individuals and richness of dung beetles abundance in four study areas in the municipalities of Bom Retiro (areas 1 and 2) and Rancho Queimado (areas 3 and 4) in the state of Santa Catarina, Brazil. Mature forest (MAF), early succession forest (ESF), Pinus monoculture (PIN) and Pastures (PAS).
(DOCX)

S2 Appendix Statistics of the generalized linear mixed model (GLMM) used to test the differences in taxonomic and functional richness between habitats with the area as a random variable. (DOCX)

S3 Appendix Environmental variables at each sampling point in four areas in the state of Santa Catarina, southern Brazil, in mature forests (FSA), early succession forest (ESF), Pinus monoculture (PIN), Pasture (PAS). (DOCX)

S4 Appendix Functional traits of 35 dung beetles species found in four areas in the state of Santa Catarina, southern Brazil. (DOCX)

S5 Appendix Abundance of functional groups in four study areas in the municipalities of Bom Retiro (area 1 and 2) and Rancho Queimado (area 3 and 4), in the state of Santa Catarina, Brazil. Mature forest (MAF), early succession forests (ESF), Pinus monoculture (PIN) and pastures (PAS).
(DOCX)
