# Supplementary material for: A decrease in taxonomic and functional diversity of dung beetles impacts the ecosystem function of manure removal in altered subtropical habitats
Source: PLoS One. 2021 Jan 6;16(1):e0244783. doi: 10.1371/journal.pone.0244783 (PMC7787441; doi:10.1371/journal.pone.0244783)
Supplement: S1 Appendix — Mature forest (MAF), early succession forest (ESF), Pinus monoculture (PIN) and Pastures (PAS). (DOCX) [file pone.0244783.s001.docx]

|  | **Area 1** | | | | **Area 2** | | | | **Area 3** | | | **Area 4** | | | |
| --- | --- | --- | --- | --- | --- | --- | --- | --- | --- | --- | --- | --- | --- | --- | --- |
| **Species** | **MAF** | **ESF** | **PIN** | **PAS** | **MAF** | **ESF** | **PIN** | **PAS** | **MAF** | **ESF** | **PAS** | **MAF** | **ESF** | **PIN** | **PAS** |
| *Canthidium* aff. *dispar* |  |  |  |  |  |  |  |  |  |  |  |  |  | 1 |  |
| *Canthidium* aff. *sulcatum* | 17 |  |  |  |  | 1 |  |  | 2 |  |  |  |  |  |  |
| *Canthidium* aff. *trinodosum* | 27 | 23 | 7 |  | 10 | 4 | 2 |  | 5 | 3 |  | 4 | 4 | 81 |  |
| *Canthidium* sp. |  |  |  |  |  |  | 1 |  |  |  |  |  |  |  |  |
| *Canthon* aff. *mutabilis* |  |  |  | 1 |  |  |  |  |  |  |  |  |  |  |  |
| *Canthon angularis* | 43 | 7 | 23 |  | 12 |  | 17 |  | 3 | 7 |  | 2 |  |  |  |
| *Canthon lividus* |  | 14 | 1 |  | 2 | 12 | 6 |  |  |  | 1 |  | 9 | 1 | 1 |
| *Canthon luctuosus* | 6 |  |  |  |  | 1 |  |  |  |  |  |  |  |  |  |
| *Canthon oliverioi* |  |  |  |  |  |  |  |  |  | 3 |  |  |  |  |  |
| *Canthon rutilans* |  |  |  |  |  |  |  |  |  | 2 |  |  | 5 | 7 |  |
| *Coprophanaeus saphirinus* | 7 | 11 | 5 |  | 5 | 9 | 7 |  | 19 | 3 |  | 9 | 9 | 8 |  |
| *Deltochilum brasiliensis* | 16 | 3 | 19 |  | 2 | 5 | 13 |  | 4 | 4 |  | 7 | 6 | 3 | 1 |
| *Deltochilum dentipes* |  |  |  |  |  |  |  |  |  |  |  |  | 2 |  | 1 |
| *Deltochilum morbillosum* |  |  |  |  |  |  |  |  | 28 | 11 |  | 15 | 5 | 6 | 1 |
| *Deltochilum multicolor* |  |  |  |  |  |  |  |  | 1 | 3 | 1 |  | 20 | 4 | 13 |
| *Deltochilum rubripenne* |  |  |  |  |  |  | 1 |  | 1 | 1 |  |  |  | 6 |  |
| *Dichotomius* aff. *acuticornis* |  |  |  |  |  |  |  |  |  | 2 |  | 4 | 4 | 3 |  |
| *Dichotomius ascanius* |  | 3 |  |  | 5 | 7 |  |  |  |  |  |  | 1 |  |  |
| *Dichotomius assifer* |  |  |  |  |  |  |  |  | 13 | 8 |  | 2 | 5 | 1 |  |
| *Dichotomius fimbriatus* |  |  |  |  |  |  |  |  |  |  |  |  | 1 |  |  |
| *Dichotomius fissus* |  |  |  |  |  |  |  |  | 1 | 2 |  |  | 1 |  |  |
| *Dichotomius mormon* |  |  |  |  |  |  |  |  |  | 1 |  | 3 | 4 | 2 | 1 |
| *Dichotomius opalescens* |  |  |  |  | 2 | 13 | 1 | 1 |  |  |  |  |  |  |  |
| *Dichotomius sericeus* |  |  |  |  |  |  |  |  | 32 | 16 |  | 24 | 29 | 44 | 20 |
| *Eurysternus cyanescens* |  |  |  |  | 1 |  |  |  | 1 | 2 |  |  |  |  |  |
| *Eurysternus inflexus* |  |  |  |  |  |  |  |  | 7 | 51 |  | 2 | 12 | 9 |  |
| *Eurysternus parallelus* |  |  |  |  |  | 3 |  |  |  |  |  |  | 1 |  | 1 |
| *Homocopris* sp. |  |  | 2 |  |  |  |  |  |  |  |  |  |  |  |  |
| *Onthophagus* aff. *hirculus* |  |  |  | 12 | 1 |  | 1 |  |  |  |  |  |  |  |  |
| *Onthophagus catharinensis* |  |  |  |  |  |  |  |  |  |  |  | 2 | 2 | 2 |  |
| *Onthophagus tristis* |  | 2 | 1 |  | 5 | 13 | 12 |  |  | 3 |  | 1 |  |  |  |
| *Phanaeus splendidulus* |  |  |  |  |  |  |  |  |  |  |  | 10 | 12 | 20 |  |
| *Uroxys dilaticollis* |  |  |  |  |  |  |  | 1 |  |  |  |  |  | 1 |  |
| *Uroxys* sp.1 | 5 | 2 | 3 |  | 5 | 3 | 12 |  | 6 | 2 |  | 24 | 31 | 50 |  |
| *Uroxys* sp.2 |  |  |  |  |  |  |  | 1 |  |  |  |  |  |  |  |
| **Total** | **121** | **65** | **61** | **13** | **50** | **71** | **73** | **3** | **123** | **124** | **2** | **109** | **163** | **249** | **39** |
