# Supplementary material for: A decrease in taxonomic and functional diversity of dung beetles impacts the ecosystem function of manure removal in altered subtropical habitats
Source: PLoS One. 2021 Jan 6;16(1):e0244783. doi: 10.1371/journal.pone.0244783 (PMC7787441; doi:10.1371/journal.pone.0244783)
Supplement: S2 Appendix — (DOCX) [file pone.0244783.s002.docx]

**Statistics of the Generalized Linear Mixed Model (GLMM) used to test the differences in taxonomic richness between habitats with the area as a random variable.**

|  | Value | Std.Error | t-value | p-value |
| --- | --- | --- | --- | --- |
| (Intercept) | 3.750 | 2.120 | 1.769 | 0.115 |
| MAF | 7.750 | 1.539 | 5.036 | 0.001 |
| ESF | 10.500 | 1.539 | 6.822 | 0.000 |
| PIN | 9.030 | 1.697 | 5.320 | 0.001 |
| Residual deviance: 2.17 on 8 degrees of freedom Number of Observations: 15 Number of Groups: 4 AIC: 72.82 | | | | |
|  |  |  |  |  |

**Statistics of the Generalized Linear Mixed Model (GLMM) used to test the differences in functional richness between habitats with the area as a random variable.**

|  | Value | Std.Error | t-value | p-value |
| --- | --- | --- | --- | --- |
| (Intercept) | 2.750 | 0.940 | 2.926 | 0.019 |
| MAF | 5.000 | 0.899 | 5.563 | 0.001 |
| ESF | 5.250 | 0.899 | 5.842 | 0.000 |
| PIN | 4.047 | 0.989 | 4.093 | 0.004 |
| Residual deviance: 1.27 on 8 degrees of freedom  Number of Observations: 15  Number of Groups: 4  AIC: 58.76 | | | | |
|  |  |  |  |  |
