# Supplementary material for: A decrease in taxonomic and functional diversity of dung beetles impacts the ecosystem function of manure removal in altered subtropical habitats
Source: PLoS One. 2021 Jan 6;16(1):e0244783. doi: 10.1371/journal.pone.0244783 (PMC7787441; doi:10.1371/journal.pone.0244783)
Supplement: S3 Appendix — (DOCX) [file pone.0244783.s003.docx]

| **Area** | **Habitat** | **Distance from the nearest tree to the center of cross** | **DBH (cm)** | **Tree height** | **Distance from the nearest shrub to the center of cross** | **DAH (cm)** | **Scrub height** | **% of leaf litter cover** | **Green cover** | **Depth of leaf litter cover** | **Temp_amb** | **Temp_sol** | **Soil density** | **Clay** | **Sand** | **Silt** | **Moisture** |
| --- | --- | --- | --- | --- | --- | --- | --- | --- | --- | --- | --- | --- | --- | --- | --- | --- | --- |
| 1 | MAF | 311.6 | 35.1 | 8.41 | 116.3 | 2.8 | 257.5 | 69.3 | 50 | 1.1 | 17 | 17.5 | 22.3 | 17.5 | 18.9 | 63.5 | 69.7 |
| 1 | ESF | 271.8 | 21.1 | 10 | 136 | 3.3 | 323 | 16.5 | 91.7 | 3 | 16.4 | 18.1 | 72.2 | 31.7 | 25.4 | 42.7 | 33.3 |
| 1 | PIN | 260 | 34.3 | 16.6 | 311.8 | 1.9 | 181.2 | 98.1 | 19.1 | 5.1 | 18.2 | 17.6 | 96.1 | 22.2 | 29.4 | 48.2 | 25.5 |
| 1 | PAS | - | - | - | - | - | - | 0 | 100 | 0 | 19.8 | 20.3 | 105.9 | 12.1 | 55.3 | 32.4 | 22.7 |
| 2 | MAF | 247.1 | 23.4 | 9.6 | 298.7 | 2.3 | 166.2 | 20.375 | 86.2 | 1.9 | 17.4 | 18.6 | 86.1 | 17.8 | 61.2 | 20.9 | 16.1 |
| 2 | ESF | 275.3 | 21.3 | 9 | 161.2 | 3.4 | 201.2 | 52.5 | 55.6 | 2.1 | 17.3 | 18.2 | 69.1 | 38.8 | 13.6 | 47.5 | 29.5 |
| 2 | PIN | 341.4 | 35 | 18 | 231.7 | 2.5 | 151.4 | 100 | 27.2 | 5.1 | 17.5 | 18.5 | 84.2 | 26.5 | 32.3 | 41.1 | 21.8 |
| 2 | PAS | - | - | - | 238.3 | 1.5 | 140 | 0 | 100 | 0 | 20.1 | 21.9 | 84.4 | 12.9 | 53.6 | 33.4 | 24.6 |
| 3 | MAF | 360 | 15.8 | 11.2 | 178 | 2.3 | 288.7 | 88.1 | 18.8 | 3.1 | 19.3 | 18.1 | 37.1 | 35.7 | 16.2 | 48 | 65.7 |
| 3 | ESF | 270 | 21.8 | 9.4 | 226.8 | 2.9 | 275 | 26 | 72.2 | 1.3 | 18.4 | 19.7 | 59.9 | 28.6 | 25.3 | 45.9 | 36.2 |
| 3 | PAS | - | - | - | - | - | - | 0 | 100 | 0 | 22 | 20.8 | 76.9 | 35.9 | 7.6 | 56.3 | 72.4 |
| 4 | MAF | 184.3 | 13.8 | 10.7 | 112.1 | 3.6 | 170 | 99.7 | 21.2 | 3.75 | 17.9 | 18.6 | 62.6 | 28.7 | 31.4 | 39.7 | 39.7 |
| 4 | ESF | 283.1 | 13.5 | 8 | 98.7 | 1.3 | 170 | 77.8 | 51.5 | 4.3 | 18.2 | 19 | 75.4 | 23.5 | 41.3 | 35 | 30.8 |
| 4 | PIN | 269.1 | 23.1 | 11.2 | 258.7 | 1.8 | 237.5 | 100 | 24.3 | 5.4 | 18 | 18.6 | 95.4 | 30.4 | 36.1 | 33.3 | 32.6 |
| 4 | PAS | 541 | 25 | 8.4 | 1500 | 0 | 0 | 17 | 97.5 | 0.5 | 19.3 | 20.5 | 111.1 | 29.8 | 35.9 | 34.2 | 32.7 |
