# Supplementary material for: A decrease in taxonomic and functional diversity of dung beetles impacts the ecosystem function of manure removal in altered subtropical habitats
Source: PLoS One. 2021 Jan 6;16(1):e0244783. doi: 10.1371/journal.pone.0244783 (PMC7787441; doi:10.1371/journal.pone.0244783)
Supplement: S4 Appendix — (DOCX) [file pone.0244783.s004.docx]

| **Species** | **Activity** | **Nesting** | **Diet** | **Biomass (g)** | **Flight capacity** | **Wing shape** | **Excavation capacity** | **Ability to roll** | **Muscle strength** |
| --- | --- | --- | --- | --- | --- | --- | --- | --- | --- |
| *Canthidium* aff. *dispar* | diurnal | paracoprid | dung | 0.010 | 4.5 | 2.7 | 0.2 | 0.3 | 2.1 |
| *Canthidium* aff. *sulcatum* | diurnal | paracoprid | generalist | 0.014 | 3.2 | 2.5 | 0.2 | 0.2 | 1.6 |
| *Canthidium* aff. *trinodosum* | diurnal | paracoprid | dung | 0.012 | 3.6 | 2.7 | 0.2 | 0.2 | 1.7 |
| *Canthidium* sp. | diurnal | paracoprid | carrion | 0.020 | 4.2 | 2.6 | 0.3 | 0.2 | 2.3 |
| *Canthon* aff. *mutabilis* | diurnal | telecoprid | dung | 0.004 | 3.0 | 2.9 | 0.3 | 0.3 | 1.5 |
| *Canthon angularis* | diurnal | telecoprid | dung | 0.037 | 5.3 | 2.7 | 0.3 | 0.3 | 2.1 |
| *Canthon lividus* | diurnal | telecoprid | p_carrion | 0.029 | 4.0 | 2.7 | 0.2 | 0.3 | 2.0 |
| *Canthon luctuosus* | nocturnal | telecoprid | p_carrion | 0.014 | 4.4 | 2.4 | 0.2 | 0.3 | 1.8 |
| *Canthon oliverioi* | diurnal | telecoprid | dung | 0.010 | 4.2 | 2.9 | 0.2 | 0.3 | 1.7 |
| *Canthon rutilans* | diurnal | telecoprid | dung | 0.051 | 4.5 | 2.8 | 0.3 | 0.3 | 2.4 |
| *Coprophanaeus saphirinus* | diurnal | paracoprid | dung | 0.342 | 8.7 | 2.5 | 0.3 | 0.2 | 5.4 |
| *Deltochilum brasiliensis* | nocturnal | telecoprid | dung | 0.045 | 12.8 | 2.6 | 0.3 | 0.5 | 5.2 |
| *Deltochilum dentipes* | nocturnal | telecoprid | dung | 0.654 | 15.9 | 2.8 | 0.2 | 0.5 | 6.1 |
| *Deltochilum morbillosum* | nocturnal | telecoprid | carrion | 0.078 | 6.2 | 2.8 | 0.2 | 0.3 | 2.7 |
| *Deltochilum multicolor* | nocturnal | telecoprid | p_dung | 0.147 | 7.0 | 2.7 | 0.2 | 0.4 | 3.6 |
| *Deltochilum rubripenne* | diurnal | telecoprid | generalist | 0.096 | 7.1 | 2.7 | 0.3 | 0.4 | 3.3 |
| *Dichotomius* aff. *acuticornis* | nocturnal | paracoprid | dung | 0.103 | 8.5 | 2.8 | 0.2 | 0.2 | 3.5 |
| *Dichotomius ascanius* | nocturnal | paracoprid | generalist | 0.067 | 8.7 | 2.5 | 0.2 | 0.2 | 3.2 |
| *Dichotomius assifer* | nocturnal | paracoprid | dung | 0.257 | 12.0 | 2.5 | 0.2 | 0.2 | 4.8 |
| *Dichotomius fimbriatus* | nocturnal | paracoprid | dung | 0.304 | 14.7 | 2.4 | 0.2 | 0.2 | 5.9 |
| *Dichotomius fissus* | nocturnal | paracoprid | dung | 0.284 | 12.4 | 2.6 | 0.2 | 0.2 | 5.3 |
| *Dichotomius mormon* | nocturnal | paracoprid | dung | 0.643 | 17.2 | 2.5 | 0.2 | 0.2 | 6.2 |
| *Dichotomius opalescens* | nocturnal | paracoprid | dung | 0.069 | 6.2 | 2.7 | 0.2 | 0.2 | 3.2 |
| *Dichotomius sericeus* | nocturnal | paracoprid | dung | 0.150 | 12.0 | 2.5 | 0.2 | 0.2 | 4.0 |
| *Eurysternus cyanescens* | nocturnal | endocoprid | generalist | 0.045 | 4.5 | 3.0 | 0.2 | 0.3 | 1.7 |
| *Eurysternus inflexus* | diurnal | endocoprid | dung | 0.021 | 3.5 | 3.4 | 0.2 | 0.3 | 1.1 |
| *Eurysternus parallelus* | diurnal | endocoprid | dung | 0.056 | 3.7 | 3.4 | 0.2 | 0.3 | 1.4 |
| *Homocopris* sp. | nocturnal | paracoprid | dung | 0.179 | 8.0 | 2.5 | 0.2 | 0.2 | 3.7 |
| *Onthophagus* aff. *hirculus* | nocturnal | paracoprid | dung | 0.007 | 2.2 | 2.8 | 0.2 | 0.2 | 1.2 |
| *Onthophagus catharinensis* | nocturnal | paracoprid | dung | 0.045 | 3.2 | 2.9 | 0.2 | 0.2 | 1.2 |
| *Onthophagus tristis* | nocturnal | paracoprid | dung | 0.012 | 2.8 | 2.9 | 0.2 | 0.2 | 1.5 |
| *Phanaeus splendidulus* | diurnal | paracoprid | dung | 0.275 | 5.8 | 2.7 | 0.2 | 0.2 | 4.7 |
| *Uroxys dilaticollis* | nocturnal | paracoprid | dung | 0.024 | 6.3 | 2.7 | 0.3 | 0.3 | 2.3 |
| *Uroxys* sp.1 | nocturnal | - | dung | 0.003 | 2.0 | 2.7 | 0.2 | 0.2 | 1.8 |
| *Uroxys* sp.2 | nocturnal | - | dung | 0.002 | 1.9 | 2.7 | 0.3 | 0.3 | 2.8 |
