# Supplementary material for: A decrease in taxonomic and functional diversity of dung beetles impacts the ecosystem function of manure removal in altered subtropical habitats
Source: PLoS One. 2021 Jan 6;16(1):e0244783. doi: 10.1371/journal.pone.0244783 (PMC7787441; doi:10.1371/journal.pone.0244783)
Supplement: S5 Appendix — Mature forest (MAF), early succession forests (ESF), Pinus monoculture (PIN) and pastures (PAS). (DOCX) [file pone.0244783.s005.docx]

|  | **Area 1** | | | | **Area 2** | | | | **Area 3** | | | **Area 4** | | | |
| --- | --- | --- | --- | --- | --- | --- | --- | --- | --- | --- | --- | --- | --- | --- | --- |
| **Functional Groups** | **MAF** | **ESF** | **PIN** | **PAS** | **MAF** | **ESF** | **PIN** | **PAS** | **MAF** | **ESF** | **PAS** | **MAF** | **ESF** | **PIN** | **PAS** |
| Group_1 | 44 | 23 | 7 |  | 10 | 5 | 3 |  | 7 | 3 |  | 4 | 4 | 82 |  |
| Group_2 | 43 | 21 | 24 | 1 | 14 | 12 | 24 |  | 4 | 13 | 1 | 2 | 14 | 14 | 1 |
| Group_3 | 6 |  |  |  |  | 1 |  |  | 28 | 11 |  | 15 | 5 | 6 | 1 |
| Group_4 |  |  |  |  | 1 |  |  |  | 1 | 2 |  |  |  |  |  |
| Group_5 |  |  |  |  |  | 3 |  |  | 7 | 51 |  | 2 | 13 | 9 | 1 |
| Group_6 | 7 | 11 | 5 |  | 5 | 9 | 7 |  | 19 | 3 |  | 19 | 21 | 28 |  |
| Group_7 |  |  | 2 |  |  |  |  |  | 46 | 27 |  | 29 | 40 | 47 | 21 |
| Group_8 | 5 | 4 | 4 | 12 | 13 | 29 | 26 | 3 | 6 | 7 |  | 31 | 37 | 56 |  |
| Group_9 |  | 3 |  |  | 5 | 7 |  |  |  |  |  |  | 1 |  |  |
| Group_10 | 16 | 3 | 19 |  | 2 | 5 | 13 |  | 4 | 4 |  | 7 | 8 | 3 | 2 |
| Group_11 |  |  |  |  |  |  |  |  | 1 | 3 | 1 |  | 20 | 4 | 13 |
